# Acid–Heat-Induced Fabrication of Nisin-Loaded Egg White Protein Nanoparticles: Enhanced Structural and Antibacterial Stability

**DOI:** 10.3390/foods13111741

**Published:** 2024-06-01

**Authors:** Shengqi Rao, Caochen Jia, Xiangning Lu, Yisheng Yu, Zhirong Wang, Zhenquan Yang

**Affiliations:** 1 College of Food Science and Engineering, Yangzhou University, Yangzhou 225127, China; sqrao@yzu.edu.cn (S.R.); chiachaochan@163.com (C.J.); 18252789453@163.com (X.L.); yuyisheng5376@163.com (Y.Y.); wangzhirongcn@163.com (Z.W.); 2 Jiangsu Key Laboratory of Zoonosis, Yangzhou 225009, China

**Keywords:** nisin, egg white protein, nanoparticle, stability of antibacterial activity, structural stability

## Abstract

As a natural cationic peptide, Nisin is capable of widely inhibiting the growth of Gram-positive bacteria. However, it also has drawbacks such as its antimicrobial activity being susceptible to environmental factors. Nano-encapsulation can improve the defects of nisin in food applications. In this study, nisin-loaded egg white protein nanoparticles (AH-NEn) were prepared in fixed ultrasound-mediated under pH 3.0 and 90 °C. Compared with the controls, AH-NEn exhibited smaller particle size (112.5 ± 2.85 nm), smaller PDI (0.25 ± 0.01), larger Zeta potential (24 ± 1.18 mV), and higher encapsulation efficiency (91.82%) and loading capacity (45.91%). The turbidity and Fourier transform infrared spectroscopy (FTIR) results indicated that there are other non-covalent bonding interactions between the molecules of AH-NEn besides the electrostatic forces, which accounts for the fact that it is structurally more stable than the controls. In addition, by the results of fluorescence intensity, differential scanning calorimetry (DSC), and X-ray diffraction (XRD), it was shown that thermal induction could improve the solubility, heat resistance, and encapsulation of nisin in the samples. In terms of antimicrobial function, acid–heat induction did not recede the antimicrobial activity of nisin encapsulated in egg white protein (EWP). Compared with free nisin, the loss rate of bactericidal activity of AH-NEn was reduced by 75.0% and 14.0% following treatment with trypsin or a thermal treatment at 90 °C for 30 min, respectively.

## 1. Introduction

Food is quite susceptible to bacterial contamination during production, transport, and distribution, which poses a great challenge to both food storage and consumer health. There are many methods to eliminate pathogenic bacteria in food, and the most traditional method is thermal sterilization technology, but the high temperature generated by this method may damage some nutrients in the food as well as bring about some bad tastes [1]. Non-thermal technologies, as a class of technologies that can inactivate pathogenic bacteria in food without heating, have also limited their application in industrialized food production due to their high cost [2,3]. As a result, antibacterial agents have become a commonly chosen method of food bacteriostasis for many food producers. Synthetic antibacterial agents are inexpensive and effective, but they are gradually being rejected by consumers because of their uncertain impact on human health [4]. Natural antibacterial agents have become a growing area of interest for food practitioners. Natural antibacterial agents are divided into three main categories, which are plant-derived antibacterial agents, animal-derived antibacterial agents, and microbial-derived antibacterial agents [5]. There have been a certain number of studies showing that natural antibacterial agents have good antibacterial effects. For example, tea polyphenols, as a plant-derived antibacterial agent widely used in food preservation, can effectively inhibit the growth of spoilage bacteria in stewed beef chunks when combined with calcium propionate [6]. Chitosan, a type of animal-derived antibacterial agent, is derived from the shells of insects and other animals, and hydrogels with antibacterial activity prepared based on it have been used in many fields, including the food industry [7].

Nisin, a type of bacteriocin produced by the *Lactococcus lactis* subspecies, is the only bacteriocin used as a food additive. As a microbial-derived antibacterial agent, it has high activity against Gram-positive bacteria like *Staphylococcus aureus* and *Listeria monocytogenes* [8]. Hence, nisin has played a great role in the food preservation field during the past decades, and studies have shown that its application has been extended to other fields such as biomedicine, as it features a broad market prospect [9]. However, the antimicrobial activity of nisin is heavily influenced by environmental factors, such as temperature, enzymes, and other conditions, and it is not able to maximize its antimicrobial activity [10,11]. This shortcoming greatly reduces the use range of nisin. At present, in order to overcome the shortcoming, liposomes, for example, are being developed as carriers for the delivery of nisin. However, the high costs of liposomes and complicated methods limit their use [12]. Recently, the use of biocompatible, biodegradable, environmentally friendly, and lipophilic nanocarriers as drug delivery systems has gained increasing attention across the world. Studies have shown that nanoparticles have the ability to protect the encapsulated active ingredients from enzyme attack, and control their release and targeted delivery [13]. Based on this advantage, the method of nanoparticles loaded with nisin can make nisin expand its application in the food industry, particularly in food preservation. Therefore, it is necessary to find a substance that is rich in resources, non-toxic, harmless, and has good biological properties, as the delivery nanocarriers of nisin.

Protein-based nanoparticles are widely used in the food industry, medicine, nutrition, and other fields due to their good characteristics [14]. Egg white protein (EWP) is regarded as an excellent protein nanocarrier because of its amphiphilicity, low toxicity, self-assembly, digestibility, rich nutritional value, and ability to interact with the encapsulated components [15]. At present, EWP has been used to prepare nanoparticles to deliver active substances such as curcumin [16], retinol [17], folic acid [18], and so on. Likewise, the protective effect of EWP on the antibacterial activity of nisin in the presence of trypsin has been reported [19]. Therefore, as a nanocarrier, EWP is an excellent choice for encapsulating nisin for nonallergic consumers. EWP can be modified in order to give it better properties. It has been documented that thermal induction opens the tertiary structure of EWP and improves its emulsifying ability and hydrophobicity [20]. Acid treatment has also been confirmed to significantly improve the functional properties of proteins [15]. However, the effect of acid–heat induction on the alteration of the properties of EWP, especially on the protective effect on nisin, is as yet unknown.

Therefore, in this study, nisin-loaded EWP nanoparticles were first prepared under acid–thermal induction, and control samples were set up. Then, the physicochemical and structural properties of the nanoparticles were evaluated by various characterization methods. Finally, the antibacterial activity of the nanoparticles and their antibacterial stability in the presence of high temperature and trypsin were determined.

## 2. Materials and Methods

### 2.1. Materials

Rongda Co., Ltd. (Xuancheng, China) provided commercial egg white protein powder from hen (moisture: ≤8.0%, purity: ≥78.0%). Nisin (≥1000 IU/mg, MW: 3354.07) and sodium 8-anilino-1-naphthalenesulfonate (ANS, 96%) were purchased from Macklin (Shanghai, China). Trypsin (1:250 from porcine pancreas) was provided by Sangon Biotech Co., Ltd. (Shanghai, China). *S. aureus* CICC 21600 was provided by the China Industrial Microbial Species Preservation and Management Center.

### 2.2. Preparation of Nisin-Loaded EWP Nanoparticles

An appropriate amount of EWP powder was weighed and completely dissolved in deionized water to prepare a 1% (*w*/*v*) EWP solution. After that, the solution was adjusted to a constant pH value at 3.0 and nisin was added to make the quantity of nisin half that of EWP powder, followed by magnetic stirring for 2 h, and then heated at 90 °C for 30 min in a water bath. Subsequently, the solution was placed immediately in an ice bath for 20 min, then transferred to a 4 °C environment overnight to form gels. The gels were minced by using an Ultrasonic Cell Disruptor (Atpio, Nanjing, China) under 600 W for 15 min and filtered by a 0.22 µm membrane to form well-distributed acid–heat-induced nisin-loaded EWP nanoparticles (AH-NEn).

Regarding the controls, the preparation methods for acid-induced nisin-loaded EWP nanoparticles (A-NEn) and acid–heat-induced EWP nanoparticles (AH-En) were essentially the same as those for AH-NEn. However, in the case of A-NEn, there was no heat treatment, and in the case of AH-En, nisin was not added.

### 2.3. Determination of Dynamic Light Scattering (DLS)

The Z-average diameter, polydispersity index (PDI), and Zeta potential of nisin, AH-En, A-NEn, and AH-NEn were measured using Malvern Zetasizer Nano (Worcestershire, UK). Nisin and the samples were diluted 20-fold with deionized water to measure Z-average diameter and PDI. During Zeta potential measurements, all samples were used without further dilution.

### 2.4. Measurement of Turbidity

The intermolecular interactions of nanoparticles can be reflected by measuring the turbidity of the samples by adding different chemical reagents. The samples were diluted four times with deionized water and then the absorbance values at 500 nm were, respectively, measured at 0 min and 30 min. At 30 min, the absorbance values of the samples at 500 nm were also measured after adding 1 M NaCl, 30 mM DTT, 0.5% SDS, and 6 M Urea [21]. The results of turbidity were represented by transmissivity.

### 2.5. Observation of Morphology

The morphology of nisin, AH-En, A-NEn, and AH-NEn was observed with a Tecnai 12 (Philips, Amsterdam, The Netherlands) at 100 kV for transmission electron microscopy (TEM) observation. The samples were dyed with a 1.5% phosphotungstic acid solution dyeing method, and put on the copper grid.

### 2.6. Fourier Transform Infrared Spectroscopy (FTIR)

The freeze-dried samples were characterized by Fourier transform infrared spectroscopy (FTIR) using a Fourier transform spectrometer (Cary 670, Agilent, Santa Clara, CA, USA) with the KBr pellet technique. Spectra were acquired by using 32 scans at 4 cm^−1^ resolution, and the scan range was from 400 to 4000 cm^−1^.

### 2.7. Determination of Fluorescence Spectra

The fluorescence spectrum of samples was acquired by using a fluorescence spectrophotometer (F-7000, Hitachi, Tokyo, Japan) to estimate their surface hydrophobicity. Before measurement, the samples were diluted with a phosphate buffer at pH 7.0 to maintain EWP concentration in all samples at 0.1% (*w*/*v*) and then mixed with 8 mM ANS solution in a 4:1 volume ratio in darkness. The excitation wavelength was 390 nm, and the emission spectral scanning range was 400 nm to 600 nm. Free nisin was also measured as a control and the concentration was consistent with that in AH-NEn.

### 2.8. Characterization of Differential Scanning Calorimetry (DSC)

The thermal properties of nisin, A-NEn, AH-En, and AH-NEn were characterized by using a differential scanning calorimetry measurement (PerkinElmer, Waltham, MA, USA). Approximately 15 mg of each sample was airtight-sealed in an aluminum pan and heated at a rate of 10 °C/min from 25 °C to 120 °C under a nitrogen flow of 20 mL/24 min.

### 2.9. Characterization of X-ray Diffraction (XRD)

The AH-En, A-NEn, AH-NEn, and nisin were characterized and compared by XRD with an X-ray diffractometer (Advance D8, Bruker, Karlsruhe, Germany) at 40 KV and 40 mA. A total of 0.2 g sample was weighed in a fixed container and placed in the instrument for measurement at room temperature. And in the scan of the diffraction pattern, a range of 4°–90° was scanned, with a step rate of 0.3 s per scan.

### 2.10. Determination of Encapsulation Efficiency and Loading Capacity

UV spectrophotometry was adopted to measure the content of nisin. Next, 400 μL sample solutions were diluted 5 times and placed in a 10 ka ultrafiltration centrifuge tube. The UV spectrophotometer (PerkinElmer, USA) was used to measure the absorbance of the filtrate at 215 nm (A_215_) and 225 nm (A_225_). The result of subtracting A_225_ from A_215_ was substituted into the linear regression equation y = 0.0002x + 0.1213 (R^2^ = 0.9990) to calculate the content of free nisin. In the equation, y is the absorbance value, x is the concentration of nisin (μg/mL), and R^2^ is the coefficient of determination. The formulas for encapsulation efficiency and loading capacity are as follows:

(1) $$\mathrm{Encapsulation}\;\mathrm{efficiency}\;(\%)=\frac{\mathrm{Total}\;\mathrm{nisin}\;\mathrm{content}\;-\;\mathrm{Free}\;\mathrm{nisin}\;\mathrm{content}}{\mathrm{Total}\;\mathrm{nisin}\;\mathrm{content}},$$

(2) $$\mathrm{Loading}\;\mathrm{capacity}\;(\%)=\frac{\mathrm{Total}\;\mathrm{nisin}\;\mathrm{content}\;-\;\mathrm{Free}\;\mathrm{nisin}\;\mathrm{content}}{\mathrm{Total}\;\mathrm{nanoparticles}\;\mathrm{content}}$$

### 2.11. Measurement of Antibacterial Activity

*S. aureus* CICC 21600 was selected as an indicator strain in antibacterial assays. Before use, the indicator strain was incubated at 37 °C for 12 h in Luria–Bertani (LB) broth to regenerate and harvested in the logarithmic phase.

The regenerated *S. aureus* CICC 21600 was diluted to an appropriate concentration for Oxford cup antibacterial experiments. An amount of 1 mL of the strain suspension was evenly coated on the agar plate. Then, Oxford cups were placed on the agar plate and the samples were added to the cup holes. The antibacterial zone diameters were measured after the samples were fully diffused at 37 °C for 12 h.

Then, 96-well microtiter plates were used to determine the minimum inhibitory concentration (MIC). First of all, ten wells were selected and marked, and then 100 µL LB broth containing the bacterial suspension diluted to 108 CFU/mL was added to each well. Negative control was made during the process of testing every sample. The MIC was determined after incubation at 37 °C for 24 h. After the testing of MIC, all samples in the unturbid test tubes were transferred to LB agar plates and incubated at 37 °C for 24 h to determine the minimum bactericidal concentration (MBC). The MBC was determined as the minimum concentration of the samples corresponding to the absence of bacterial growth in the LB agar plate.

The samples were, respectively, treated with 121 °C and 5 mg/mL of trypsin for 15 min, and then the test of determination of MIC and MBC was repeated to observe their antibacterial stability. The final results were expressed as the antibacterial efficacy loss rate.

### 2.12. Statistical Analysis

The Origin 8.5 and IBM SPSS Statistics 25 software programs were used for all statistical analyses. All the tests were performed in triplicate to confirm reproducibility. It was considered statistically significant when the *p* value was less than 0.05 according to Duncan’s multiple range test.

## 3. Results and Discussion

### 3.1. Z-Average Diameter, PDI, and Zeta Potential

pH and heat treatment conditions can significantly affect particle size and binding capacity to hydrophobic functional components of protein nanovehicles produced from EWP [22]. In addition, suitable ultrasonic conditions can help egg white protein aggregate or colloidal particles to form small particle sizes and a stable structure of nanoparticles [23]. Therefore, EWP nanoparticles loaded with nisin were prepared using acid–heat induction, followed by an ultrasonic treatment (15 min of 3 s on/off pulses at 600 W), and the changes in physicochemical properties, structural properties and antibacterial properties before and after thermal treatment, and nisin-loaded nanoparticles were investigated in this study. From Table 1, it can be seen that the Z-average diameter, PDI, and Zeta potential of the nisin solution and three different samples. The prepared samples are all at the nanoscale, with AH-NEn having the smallest Z-average diameter (112.5 nm). The smallest Z-average diameter of AH-NEn was probably caused by the combination of ultrasonic thermal induction and invasion of nisin. Generally, a PDI below 0.3 signifies a high degree of uniformity in the sample dispersion [24]. Therefore, from the PDI results, it can be seen that only the dispersion of AH-NEn with a PDI value of 0.25 is acceptable. At a pH of 3.0, both nisin and EWP are positively charged. When nisin and EWP nanoparticles combined, the Zeta potential significantly increased, indicating the existence of electrostatic force between the two [25]. The higher Zeta potential observed in AH-NEn (24 mV) as compared to A-NEn (18.5 mV) may be attributed to structural changes in the protein induced by heat, leading to the exposure of additional amino acid residues on the surface. Previous studies have shown that the absolute value of potential is positively correlated with the stability of nanoparticles [26]. So, the Zeta potential results also indicate that the stability of AH-NEn is better than the other two.

### 3.2. Analysis of Intermolecular Interaction Force

**Figure 1:**
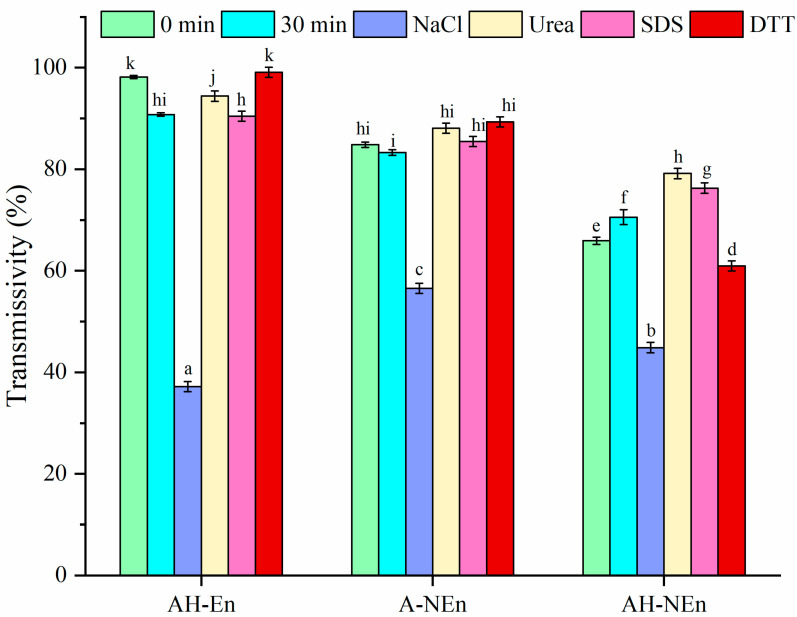
The influence of NaCl, Urea, SDS, and DTT on the turbidity of nisin, EWP nanoparticles, and nisin-loaded EWP nanoparticles. Different superscript letters indicate statistically significant differences (*p* < 0.05).

NaCl, Urea, SDS, and DTT are, respectively, utilized to disrupt ion interactions, hydrogen bonding, disulfide bonding, and hydrophobic interaction [21]. As shown in Figure 1, the transmissivity of AH-En, A-NEn, and AH-NEn decreased significantly after the addition of NaCl at 30 min. This observation suggests that all three samples exhibit electrostatic forces, which aligns with the findings of the Zeta potential measurements. Among the samples, AH-En is the most affected by electrostatic forces, indicating minimal or no shielding of the surface charge of EWP. The transmissivity of A-NEn is primarily influenced by NaCl, suggesting the prevalence of electrostatic interactions within A-NEn. The transmissivity of AH-NEn is affected by NaCl and DTT, indicating the presence of electrostatic force and disulfide bonding. This is due to the thermal induction promoting the generation of new intermolecular interaction forces between EWP and nisin.

### 3.3. Morphological Structure Analysis

**Figure 2:**
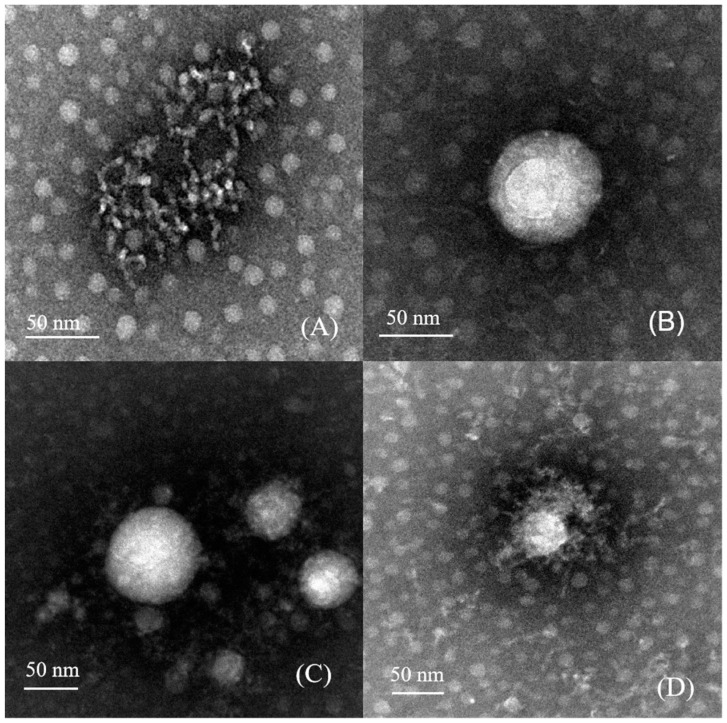
TEM images of nisin (**A**), acid–heat-induced EWP nanoparticles (**B**), acid-induced nisin-loaded EWP nanoparticles (**C**), and acid–heat-induced nisin-loaded EWP nanoparticles (**D**).

In Figure 2, TEM images of the samples are shown to observe their surface morphology. Except for loosely aggregated nisin, spherical particles can be clearly observed in all protein nanoparticles. Nisin demonstrates a chain-like structure, which is observed to either surround or be interspersed within the nanoparticles. It can be seen from Figure 2C,D that AH-NEn has stronger aggregation than A-NEn, which is the result of intermolecular forces strengthening after heat treatment. However, it is worth noting that the particles observed via TEM are smaller in size compared to those measured by the Malvern Zetasizer Nano (Table 1). This discrepancy may be caused by the expansion of biopolymer nanoparticles in aqueous solutions, or by differences in calculation methods and measurement systems [27].

### 3.4. FTIR Analysis of Nisin-Loaded EWP Nanoparticles

**Figure 3:**
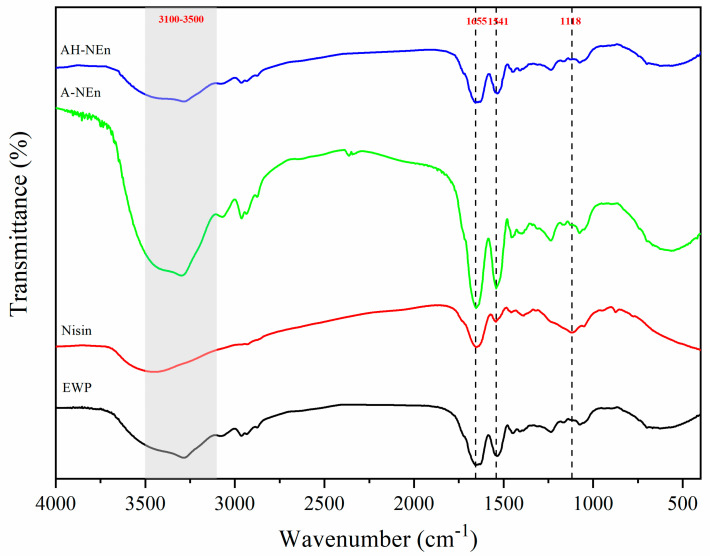
FTIR spectra of nisin, EWP, and nisin-loaded EWP nanoparticles.

In the spectral range of 3100–3500 cm^−1^, the observed peak shift can provide insights into the formation or alteration of hydrogen bonds, as these vibrations were caused by axial O-H and N-H tensile vibrations [28]. Nisin demonstrated distinctive absorption peaks at 1655 cm^−1^ (amide I), 1541 cm^−1^ (amide II), and 1118 cm^−1^ (amide III) [29,30]. Figure 3 shows that nisin, EWP, A-NEn, and AH-NEn had broad characteristic peaks at 3466 cm^−1^, 3294 cm^−1^, 3300 cm^−1^, and 3284 cm^−1^. And the O-H and N-H stretching bands in nisin (3466 cm^−1^) and EWP (3294 cm^−1^) considerably shifted to 3300 cm^−1^ in A-NEn and 3284 cm^−1^ in AH-NEn, respectively. This phenomenon can be attributed to the formation of strong hydrogen bonds between nisin and EWP during the particle formation process induced by acid or acid–heat conditions. [31,32]. Meanwhile, the amide bands in the above two nanoparticles moved (amide I from 1653 to 1657 cm^−1^, amide II from 1541 to 1535 cm^−1^, and amide III from 1118 to 1074 cm^−1^) after the nisin was incorporated into EWP under acid or acid–heat induction conditions. In addition, the intensity of the band at 1118 cm^−1^ decreased after the interaction of nisin with EWP for particle formation. These changes indicate an electrostatic interaction between the two substances [29,33]. Compared with A-NEn, the N-H stretching band and amide band absorption peaks of AH-NEn showed a much larger shift, indicating that heat treatment further promotes the formation of hydrogen bonds and electrostatic interaction of acid-induced nisin–EWP nanoparticles.

### 3.5. Fluorescence Spectra Analysis of Nisin-Loaded EWP Nanoparticles

**Figure 4:**
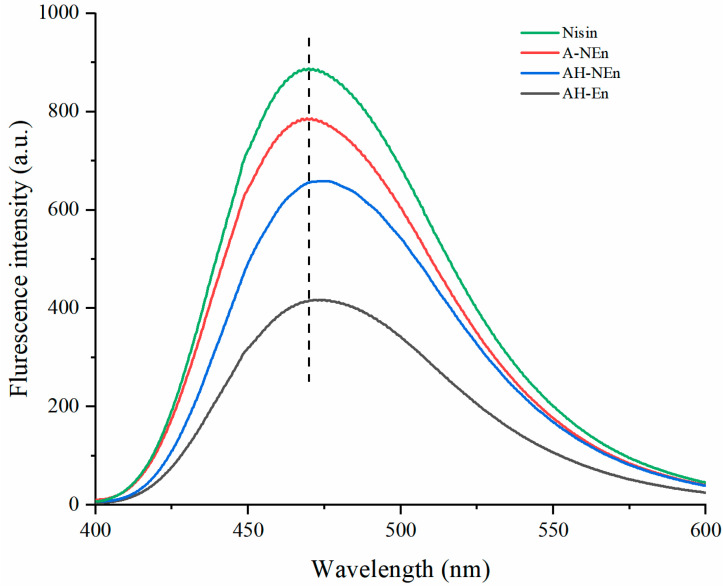
The fluorescence spectra of nisin, EWP nanoparticles, and nisin-loaded EWP nanoparticles.

The prevailing belief suggests a positive correlation between fluorescence intensity and surface hydrophobicity [34]. Analysis of Figure 4 indicates that the surface hydrophobicity of the measured samples, from strong to weak, is as follows: nisin, A-NEn, AH-NEn, and AH-En. Nisin is positively charged under acidic conditions and ANS is negatively charged [35]. This could facilitate the combination of nisin with ANS with a negative charge, resulting in the strongest surface hydrophobicity of free nisin [36]. Compared with AH-En, the enhanced surface hydrophobicity of AH-NEn is due to the enhanced intermolecular interactions between the nanoparticles after the addition of nisin, which alters the protein structure and exposes the hydrophobic groups inside the protein [37]. The lower surface hydrophobicity of AH-En compared to A-NEn may be due to the destruction of some surface hydrophobic groups by heat treatment. The stronger the surface hydrophobicity, the higher the emulsibility, and the poorer the solubility [38]. Consequently, AH-NEn exhibits superior emulsifying capabilities relative to AH-En and enhanced solubility compared to A-NEn.

### 3.6. DSC Analysis of Nisin-Loaded EWP Nanoparticles

**Figure 5:**
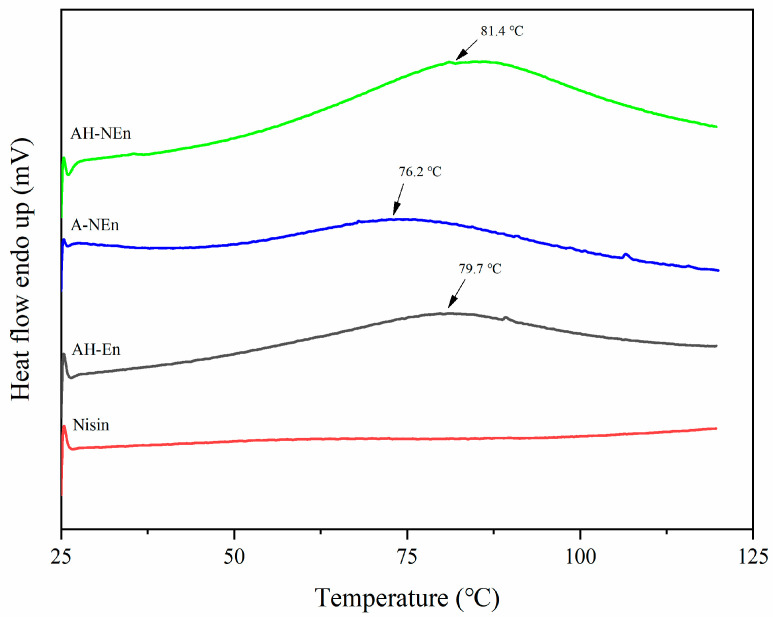
DSC curves of nisin, EWP nanoparticles, and nisin-loaded EWP nanoparticles under nitrogen atmosphere. Samples were scanned from 25 °C to 120 °C at a heat rate of 10 °C/min.

The DSC curves of AH-NEn, A-NEn, AH-En, and nisin are presented in Figure 5. From the figure, we can clearly see that all of the samples showed a broad endothermic peak between 70 °C and 90 °C, which is likely to indicate that ovalbumin unfolded [39,40]. Compared with AH-En (Td = 79.7 °C), the denaturation temperature of the EWP in the acid–heat-induced nisin-loaded EWP nanoparticles AH-NEn was slightly enhanced (Td = 81.4 °C), and it is suggested that proteins with nisin are more resistant to heat. However, compared with AH-NEn, the Td value of A-NEn reduced to 76.2 °C. The above results suggested that the addition of nisin and heat treatment could enhance the thermal stability of acid-induced EWP nanoparticles.

### 3.7. XRD Analysis of Nisin-Loaded EWP Nanoparticles

**Figure 6:**
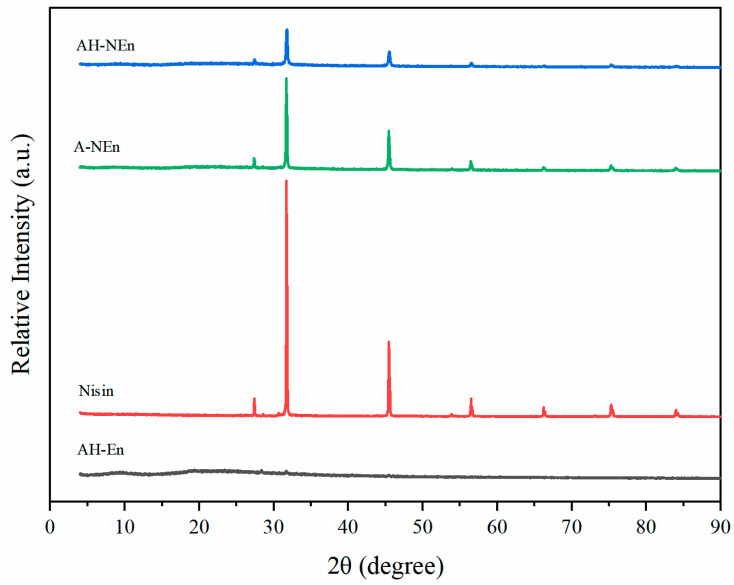
XRD patterns of nisin, EWP nanoparticles, and nisin-loaded EWP nanoparticles.

The XRD patterns of EWP, nisin, and nisin–EWP nanoparticles are shown in Figure 6. No evident peaks were observed for EWP, reflecting the amorphous nature of EWP. An XRD analysis of nisin powder revealed five distinctive peaks at 27.37°, 31.72°, 45.46°, 53.87°, and 56.49° due to sodium chloride diffraction patterns, which are similar to other reports [27,41]. There were no alterations observed in the characteristic peaks for A-NEn and AH-NEn, suggesting that the resultant particles maintained the crystalline structure of nisin without compromising its integrity. However, nisin–EWP microcapsules exhibit a significantly diminished diffraction pattern than nisin alone. A decrease in crystallinity might be caused by the encapsulation of nisin within EWP nanoparticles. In contrast to A-NEn, AH-NEn had lower peak intensities in its diffraction pattern, indicating that more nisin was encapsulated into AH-NEn. The increased heating of AH-NEn may have facilitated stronger interactions between EWP and nisin, leading to a reduction in peak intensity. In order to explain this phenomenon, we may need to consider the following: heating promoted more nisin molecules dispersed in the EWP nanoparticle matrix and inhibited the aggregation of their crystals, thus promoting the formation of more amorphous complexes. Similar results have also been reported for biopolymer particles, which were formed by the heating of β-lactoglobulin–gum arabic complexes in the presence of epigallocatechin-3-gallate with enhanced encapsulation efficiency [42]. These findings suggest that the heating process promoted additional noncovalent interactions between EWP and nisin, ultimately enhancing the encapsulation of nisin within EWP nanoparticles.

### 3.8. Encapsulation Capacity of EWP Nanoparticles

**Figure 7:**
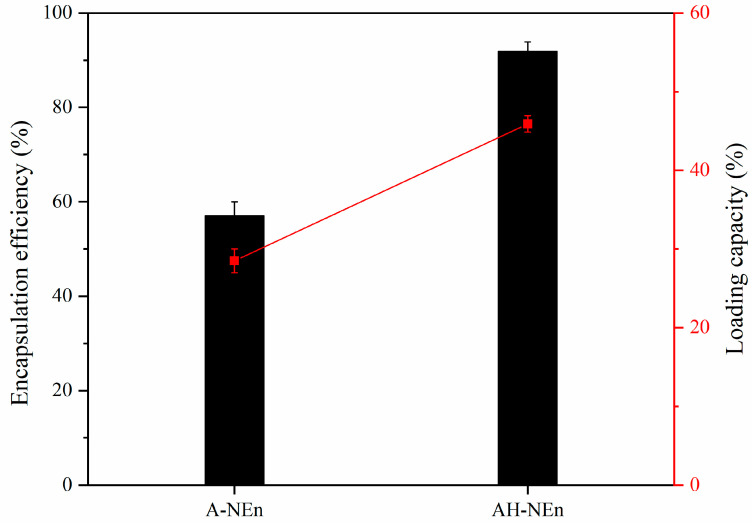
Encapsulation efficiency and loading capacity of nisin-loaded EWP nanoparticles. Values are means ± standard deviations, indicated by error bars.

The encapsulation capacity of the two nanoparticles, A-NEn and AH-NEn, was evaluated by measuring the encapsulation efficiency and loading capacity, as shown in Figure 7. From the figure, it can be seen that AH-NEn has an encapsulation efficiency of 91.82% and a loading capacity of 45.91%, both of which are twice as high as A-NEn. This suggests that thermal induction can significantly enhance the encapsulation efficiency of EWP nanoparticles. This phenomenon may be related to the fact that thermal induction can alter the protein structure, making the interior of the protein more stretched [43]. Therefore, due to the ability to load more nisin, AH-NEn is more efficient than A-NEn in practical applications.

### 3.9. Antibacterial Properties Analysis of Nisin-Loaded EWP Nanoparticles

**Figure 8:**
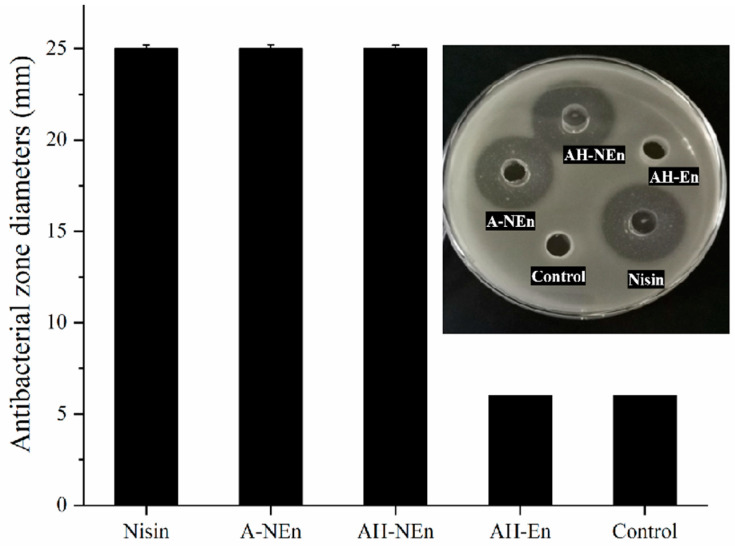
Antibacterial zone diameters of nisin, EWP nanoparticles, and nisin-loaded EWP nanoparticles. Values are means ± standard deviations, indicated by error bars.

The antibacterial zone diameters of A-NEn, AH-En, AH-NEn, and nisin shown in Figure 8 can intuitively reflect their antibacterial activity. The results showed that AH-En did not exhibit antibacterial activity compared to the control. The antibacterial activity of nisin is responsible for the similar antibacterial zone diameters observed for nisin, A-NEn, and AH-NEn, all measuring around 25 mm with no significant variance.

Before the measurement, based on the results of encapsulation capacity, AH-NEn and A-NEn were accurately weighed and the initial concentration of nisin was maintained at 5 mg/mL. Table 2 shows the MIC and MBC of nisin, A-NEn, and AH-NEn were all 156.25 µg/mL and 312.5 µg/mL, respectively. Therefore, nisin, A-NEn, and AH-NEn had consistent antibacterial activities. In addition, the results also indicate that the high temperature and acidic conditions during the preparation of nanoparticles did not affect the antibacterial activity of nisin. There are two potential explanations for this. Firstly, nisin exhibits notable thermal stability due to its high content of heat-stable compounds resulting from the linkage of two amino acids through disulfide bonds. Secondly, the structure of nisin was not destroyed during the preparation process. The XRD results (Figure 6) show that the structural integrity of nisin is retained in both A-NEn and AH-NEn. Previous studies have suggested that the combination of nisin with some polymer materials may alter its spatial structure, thereby reducing the antibacterial activity of nisin [44]. For example, a study has shown that gum arabic cross-link binding nisin microparticles have lower antibacterial properties compared with those of nisin alone [45]. However, in this study, the combination of EWP with nisin did not affect the antibacterial activity of nisin. So, as a material loaded with nisin, EWP has more advantages than some other polymer materials.

Trypsin, a digestive enzyme found in meat products, has been shown to destabilize the biological activity of nisin. In order to verify the antibacterial stability of nisin-loaded EWP nanoparticles, the loss rate of bactericidal activity of free nisin and nisin-loaded EWP nanoparticles under trypsin and high-temperature conditions were tested, respectively, and the results are shown in Table 3. Under the action of trypsin, the antibacterial activity of nisin almost disappears, while the antibacterial activity of A-NEn remains almost unchanged, and the antibacterial activity of AH-NEn only loses 12.5%. This indicates that the combination with EWP can significantly enhance the resistance of nisin to trypsin. The protection of nisin antibacterial activity by EWP has also been reported in the previous literature [19]. This is because EWP prevents full contact between trypsin and nisin, protecting nisin from trypsin degradation [10]. Furthermore, EWP contains ovomucoid, a trypsin inhibitor that enables EWP to effectively protect nisin [46]. It should be noted that the loss rate of bactericidal activity of AH-En is slightly higher than that of A-NEn. This phenomenon may be due to the changes in the structure of EWP caused by the high-temperature conditions during the preparation of AH-NEn, thereby reducing the inhibitory effect of EWP on trypsin. Anyway, the antibacterial activity of AH-NEn in trypsin still retained 87.5%. The previous literature has shown that heating can reduce the antibacterial activity of nisin, which is also shown in Table 3 [47]. Under high-temperature conditions, the loss rate of bactericidal activity of nisin-loaded nanoparticles is smaller than that of free nisin, indicating that EWP can also improve the antibacterial thermal stability of nisin. And Table 3 shows that the antibacterial activity of AH-NEn under high-temperature conditions is significantly better than that of A-NEn. This is due to the heat treatment during the preparation process, which enables AH-NEn to have a more compact structure, thereby enabling nisin to maintain better biological activity. This is consistent with the DSC analysis results (Figure 5). In summary, encapsulation of nisin in EWP nanoparticles under acidic conditions has been found to significantly enhance its antibacterial activity in the presence of trypsin. Additionally, thermal induction can further enhance the antibacterial activity of nisin at high temperatures.

## 4. Conclusions

The aim of this research is to prepare EWP nanoparticles loaded with nisin, followed by the characterization of their physicochemical properties and assessment of their antibacterial efficacy. AH-NEn, AH-En, and A-NEn nanoparticles were successfully prepared under conditions of pH 3.0 at 90 °C. The findings of this study revealed that AH-NEn nanoparticles exhibit additional noncovalent interactions, alongside electrostatic forces, resulting in a more stable and compact structure compared to the other nanoparticle variants. Furthermore, among the three samples, AH-NEn exhibits the smallest Z-average diameter, superior thermal stability, and optimal encapsulation of nisin. In terms of antibacterial function, the antibacterial activity of nanoparticles mainly comes from nisin. EWP, serving as an encapsulation material, does not possess inherent antibacterial activity; it significantly enhances the antibacterial activity of nisin in trypsin. The acidic conditions and thermal induction utilized during the preparation of AH-NEn do not diminish the antibacterial activity of nisin, but can also enhance its antibacterial activity under high-temperature conditions. Moreover, the process of thermal induction significantly enhances the encapsulation efficiency of EWP nanoparticles. In conclusion, AH-NEn exhibits promising potential as a natural antibacterial agent with strong antibacterial properties, and its application range is wider than free nisin.

## Figures and Tables

**Table 1:** Z-average diameter, PDI, and Zeta potential of nisin, EWP nanoparticles, and nisin-loaded EWP nanoparticles.

Sample	Z-Average Diameter (nm)	PDI	Zeta Potential (mV)
Nisin	760.00 ± 4.55 ^d^	0.86 ± 0.06 ^d^	7.45 ± 0.98 ^a^
AH-En	231.90 ± 6.01 ^c^	0.51 ± 0.03 ^b^	15.60 ± 1.89 ^b^
A-NEn	184.30 ± 5.73 ^b^	0.73 ± 0.05 ^c^	18.50 ± 2.01 ^c^
AH-NEn	112.50 ± 2.85 ^a^	0.25 ± 0.01 ^a^	24.00 ± 1.18 ^d^

Values represent the means ± SD (*n* = 3). Different superscript letters in the same column indicate statistically significant differences (*p* < 0.05).

**Table 2:** Minimum inhibitory concentration (MIC) and minimum bactericidal concentration (MBC) against *S. aureus* CICC 21600.

Sample	MIC (μg/mL)	MBC (μg/mL)
Nisin	156.25	312.50
A-NEn	156.25	312.50
AH-NEn	156.25	312.50

The tests were conducted with the initial concentration of nisin in all samples kept at 5 mg/mL.

**Table 3:** The loss rate of bactericidal activity of nisin and nisin-loaded EWP nanoparticles on *S. aureus* CICC 21600 after tryptic digestion and after 121 °C treatment for 15 min.

Treatment	Loss Rate of Bactericidal Activity (%)		
	Nisin	A-NEn	AH-NEn
Tryptic digestion	87.50 ± 0.89 ^c^	2.50 ± 1.52 ^a^	12.50 ± 0.96 ^b^
Heat treatment	31.25 ± 1.12 ^c^	28.12 ± 1.33 ^b^	17.50 ± 1.03 ^a^

Values represent the means ± SD (*n* = 3). There are statistically significant differences (*p* < 0.05) between the two columns that are indicated with different superscript letters. The tests were conducted with the final concentration of nisin in all samples kept at 5 mg/mL.

## Data Availability

The original contributions presented in the study are included in the article, further inquiries can be directed to the corresponding author.
